# Attenuated Toxicity and Antitoxic Mechanism via Sodium Iodide Symporter Inhibition-Based Tumor-Selective Delivery in Astatine-211 Radioimmunotherapy

**DOI:** 10.1021/acs.molpharmaceut.5c01438

**Published:** 2026-03-01

**Authors:** Hiroki Takashima, Ryo Tsumura, Yoshikatsu Koga, Takahiro Anzai, Xiaojie Yin, Nozomi Sato, Yudai Shigekawa, Yousuke Kanayama, Akihiro Nambu, Sachiko Usuda, Hiromitsu Haba, Shingo Sakashita, Anri Inaki, Shino Manabe, Masahiro Yasunaga

**Affiliations:** † Division of Developmental Therapeutics, 581202 Exploratory Oncology Research & Clinical Trial Center, National Cancer Center, 6-5-1 Kashiwanoha, Kashiwa, Chiba 277-8577, Japan; ‡ Nishina Center for Accelerator-Based Science, 13593 RIKEN, 2-1 Hirosawa, Wako, Saitama 351-0198, Japan; § Division of Pathology, 581202 Exploratory Oncology Research & Clinical Trial Center, National Cancer Center, 6-5-1 Kashiwanoha, Kashiwa, Chiba 277-8577, Japan; ∥ Division of Functional Imaging, 581202 Exploratory Oncology Research & Clinical Trial Center, National Cancer Center, 6-5-1 Kashiwanoha, Kashiwa, Chiba 277-8577, Japan; ⊥ Laboratory of Synthetic Biomolecular Chemistry, School of Pharmacy and Pharmaceutical Sciences and Institute of Medical Chemistry, 34764 Hoshi University, 2-4-41 Ebara, Shinagawa-ku, Tokyo 142-8501, Japan; # Research Center for Pharmaceutical Development, Graduate School of Pharmaceutical Sciences & Faculty of Pharmaceutical Sciences, Tohoku University, 6-3 Aoba, Aramaki, Aoba-ku, Sendai 980-8578, Japan; ¶ Glycometabolic Biochemistry Laboratory, 13593 RIKEN, 2-1 Hirosawa, Wako, Saitama 351-0198, Japan

**Keywords:** astatine-211, tumor-selective delivery, coupling reagent, sodium iodide symporter inhibition, toxicity

## Abstract

Astatine-211 (^211^At) is a promising alpha emitter for cancer treatment, wherein tumor-selective accumulation is pivotal due to its short path length. While sodium ascorbate (SA) successfully protects radioactive antibodies from reactive oxygen species (ROS)-induced denaturation, it does not reduce ^211^At distribution in normal organs or mitigate body weight loss. Here, we aimed to attenuate this normal organ uptake. We demonstrated that sodium perchlorate (SP), a competitive inhibitor of the sodium iodide symporter (NIS) expressed in thyroid and gastric mucosal cells, significantly reduced ^211^At uptake in the stomach and thyroid in ^211^At-radioimmunotherapy (RIT) under SA protection. This favorable biodistribution resulted in significantly milder body weight loss without attenuating the antitumor effect. The combined strategy proved feasible, with no renal toxicity and no exacerbation of transient hematotoxicity or hepatotoxicity. Crucially, NIS inhibition significantly reduced DNA double-strand breaks in stomach and thyroid tissues and helped maintain the thyroid’s follicular structure. Overall, we demonstrate that combining SA protection to prevent antibody denaturation with competitive NIS inhibition by SP for greater tumor-selective ^211^At delivery is feasible, broadens the therapeutic window, and facilitates the clinical application of ^211^At-RIT in cancer treatment.

## Graphical abstract

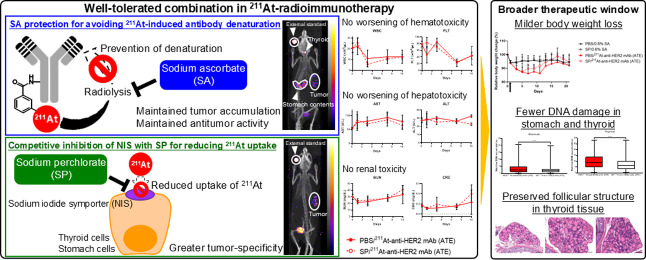

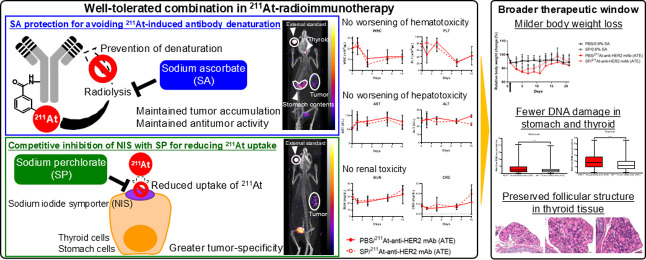

## 1 Introduction

Alpha particles are characterized by a high linear energy transfer, which results in efficient double-strand breaks (DSBs) in DNA, and a short path length of 50–100 μm in tissues.^1^ Thus, tumor-selective accumulation of alpha emitters is pivotal for achieving reasonable efficacy and safety in targeted alpha therapy. Astatine-211 (^211^At) is an alpha-emitting halogen applicable for cancer treatment. As a therapeutic radionuclide, ^211^At has the following four favorable properties: radionuclide has a half-life of 7.2 h, which is sufficiently long to prepare radioactive pharmaceuticals through radioisotope (RI) labeling.^2^ Second, a cyclotron can produce a high yield of ^211^At, rendering it feasible to prepare astatinated pharmaceuticals with clinically effective radioactivity doses.^2^ Third, ^211^At decays through double-branched pathways and produces one alpha particle per pathway, resulting in 100% alpha emissions.^2^ Fourth, besides alpha particles, ^211^At emits characteristic X-rays that allow the imaging and evaluation of the in vivo biodistribution of astatinated pharmaceuticals using planar or single-photon emission computed tomography (SPECT).^3–5^

Antibodies are available as drug carriers for tumor-selective ^211^At delivery, and various target molecules are suitable for radioimmunotherapy (RIT) with alpha emitters.^5–12^ In the RI labeling process for preparing radioactive antibodies, careful attention should be paid to issues caused by radionuclide-induced chemical reactions, such as disrupted specific binding activity.^13–17^ We previously demonstrated that reactive oxygen species (ROS), generated through the radiolysis of water by monoclonal antibodies (mAbs) labeled with ^211^At (^211^At-mAbs), induce denaturation of the radioactive mAbs themselves. By adding sodium ascorbate (SA) to the storage solution, the free radical scavenger can quench ROS and prevent ^211^At-mAbs from denaturing in a concentration-dependent manner.^18^ Although the ^211^At-induced radiochemical reaction disrupts the specific binding of radioactive mAbs to cancer cells, the binding activity of SA-stabilized astatinated mAbs is comparable to that of unmodified antibodies.^5,11,18^ Moreover, in vivo tumor accumulation of ^211^At-mAbs under SA protection was significantly higher than that of radioactive mAbs denatured by ROS.^5^ Consistently, astatinated mAbs stabilized with SA showed significantly greater antitumor effects than denatured radioactive mAbs.^5,11^ Therefore, in ^211^At-RIT, SA protection can prevent ROS-induced antibody denaturation and maintain the binding activity, tumor accumulation, and antitumor effect of astatinated mAbs. However, no difference was observed in ^211^At distribution in normal organs or body weight loss after RIT between the groups treated with ^211^At-mAb under SA protection and denatured radioactive mAb.^5,11^

Reducing the ^211^At distribution in normal organs is expected to contribute to lower toxicity. In this study, we introduce two strategies to reduce radiation exposure in normal tissue. First, we evaluated two coupling reagents available to prepare ^211^At-mAbs, *N*-succinimidyl-3-(trimethylstannyl)­benzoate (ATE) and *N*-[2-(maleimido)­ethyl]-3-(trimethylstannyl)­benzamide (MSB), and determined the better reagent in terms of less ^211^At release from radioactive antibodies and tumor-selective distribution. Similar to iodine, which is a halogen, free ^211^At accumulates in the stomach and thyroid.^4,19^ Thus, a coupling reagent that stably maintains ^211^At on antibodies is expected to reduce ^211^At release and the radiation dose to the stomach and thyroid. Using ATE or MSB, Aneheim et al. prepared ^211^At-mAbs and characterized their radioactive immunoconjugates in terms of tumor accumulation and distribution in normal organs.^20^ However, ^211^At liberation and the biodistribution of these ^211^At-labeled immunoconjugates under SA protection have not been clarified. Second, we reduced stomach and thyroid radiation doses using sodium perchlorate (SP), a competitive inhibitor of the sodium iodide symporter (NIS), and evaluated the antitoxic activity of ^211^At-RIT. NIS is a plasma membrane glycoprotein expressed in thyroid cells.^21–24^ Iodine, a halogen, is transported via NIS, and its oxidized form is incorporated into thyroglobulin and stored in the thyroid colloid.^21,22^ In contrast, NIS expressed in gastric mucosal cells contributes to the concentration of iodine and its subsequent secretion into gastric juices.^21,22,24–26^ Besides iodine, ^211^At is also a halogen. Thus, the fact that ^211^At accumulated in the stomach and thyroid is reasonable.^4,19^ Although competitive inhibitors of NIS can reduce the uptake of ^211^At in the thyroid and stomach,^27,28^ the antitoxic effects of tumor-selective delivery via NIS inhibition in ^211^At-RIT remain unclear. In this study, we aimed to elucidate the tolerability and antitoxic activity of SP in ^211^At-RIT under SA protection conditions.

## 2 Materials and Methods

### 2.1 Antibody

Trastuzumab, an mAb that recognizes human epidermal growth factor receptor 2 (HER2), was purchased from Chugai Pharmaceuticals (Tokyo, Japan).

### 2.2 Gastric Cancer Cell Lines

The human gastroesophageal junction cancer cell line OE19 and human gastric cancer cell line NUGC-3 were purchased from the European Collection of Authenticated Cell Cultures (ECACC; London, UK) and the Japanese Collection of Research Bioresources (JCRB; Osaka, Japan), respectively. The cells were cultured in RPMI-1640 medium (FUJIFILM Wako Pure Chemical Corporation, Osaka, Japan) supplemented with 10% fetal bovine serum (Thermo Fisher Scientific, Waltham, MA, USA), 100 units/mL penicillin, 100 μg/mL streptomycin, and 0.25 μg/mL amphotericin B (FUJIFILM Wako Pure Chemical Corporation) at 37 °C in a humidified 5% CO_2_ atmosphere.

### 2.3 Animal Model

To prepare the model mice, 3 × 10^6^ OE19 cells suspended in 100 μL of phosphate-buffered saline (PBS) were inoculated into the flank region of six-week-old female BALB/c nu/nu mice (Charles River Japan, Yokohama, Japan). Tumor volume was calculated using the following formula: tumor volume = (length × width^2^) × 1/2. Animal experiments were approved by the Committee for Animal Experimentation of the National Cancer Center in Japan (K21-003, K21-003-M01, and K24-005). All animal procedures were performed in compliance with the Guidelines for the Care and Use of Experimental Animals established by the Committee. These guidelines met the ethical standards required by law and complied with the guidelines to use experimental animals in Japan.

### 2.4 ^211^At Production

^211^At was produced through the ^209^Bi­(α,2n)^211^At reaction using the RIKEN AVF cyclotron (RIKEN, Wako, Japan) and prepared solid ^211^At.^11^

### 2.5 Preparation of ^211^At-mAbs

ATE was synthesized as described previously.^29^ Trastuzumab conjugated with ATE (Sn-anti-HER2 mAb (ATE)) was prepared according to a previously described procedure with minor modifications (Figure 1A).^20,30^ Briefly, the storage solution for trastuzumab was changed to 0.2 M sodium bicarbonate (pH 8.5) by ultrafiltration, and the antibody concentration was adjusted to 4 mg/mL. After buffer exchange, the antibodies were incubated with a 5-fold molar excess of ATE for 30 min. The buffer solution was then replaced with PBS, and unconjugated ATE molecules were removed. The ATE-immunoconjugate was stored at −80 °C until use.

**Figure 1:**
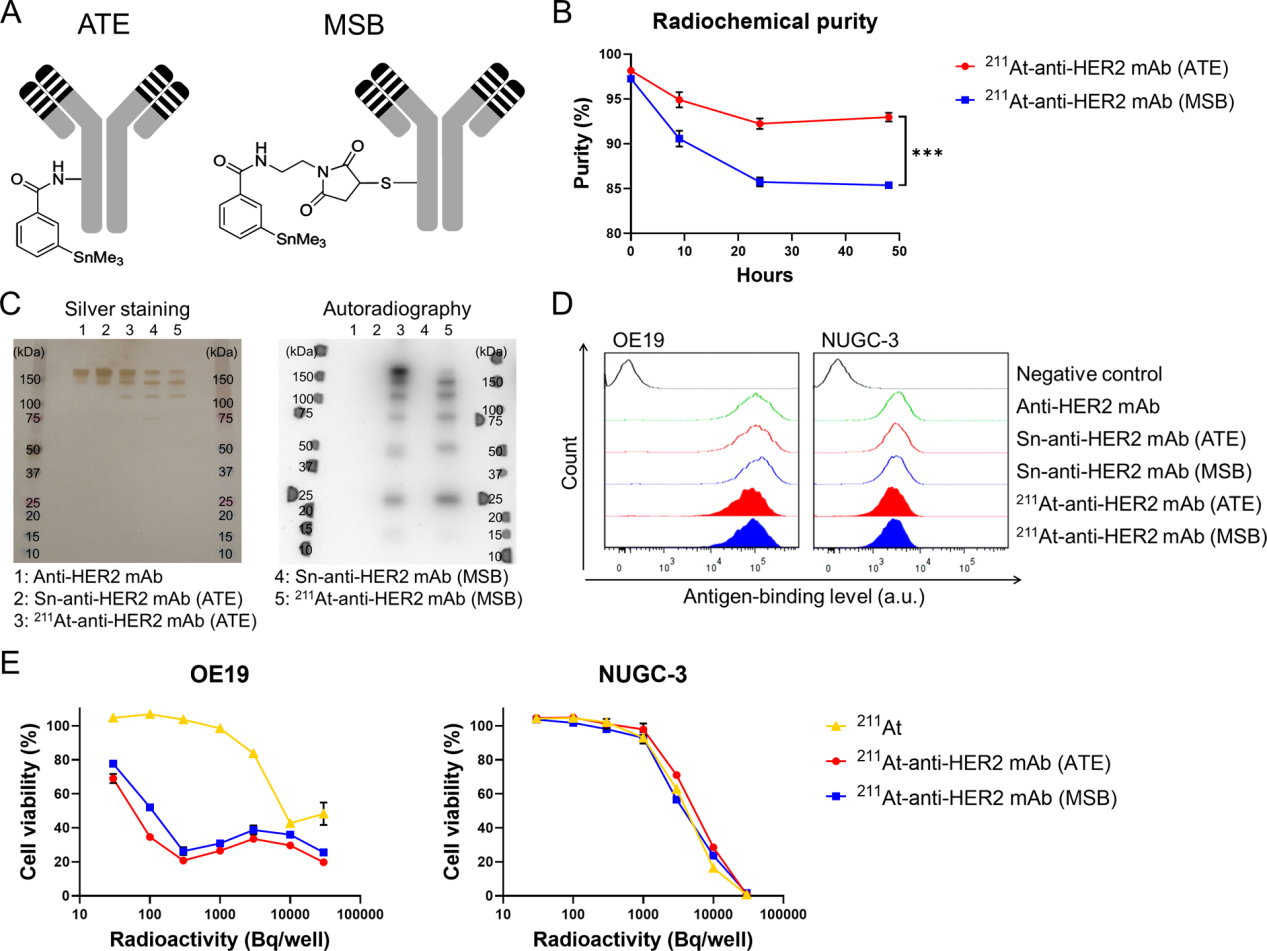
Characterization of ^211^At-anti-HER2 mAbs. (A) Diagram of antibodies conjugated to ATE or MSB. ATE and MSB were *N*-succinimidyl-3-(trimethylstannyl)­benzoate and *N*-[2-(maleimido)­ethyl]-3-(trimethylstannyl)­benzamide, respectively. (B) Radiochemical purity and its temporal change. Both ^211^At-anti-HER2 mAb (ATE) and ^211^At-anti-HER2 mAb (MSB) were purified in PBS containing 0.6% SA and stored at 4 °C. The ^211^At liberation in the ^211^At-anti-HER2 mAb (ATE) solution was significantly lower than that in the ^211^At-anti-HER2 mAb (MSB) solution (*P* < 0.001) (*n* = 3). Points represent the mean, and bars represent the standard deviation. (C) Sodium dodecyl sulfate-polyacrylamide gel electrophoresis (SDS-PAGE) analysis. ^211^At-mAbs eluted in PBS containing 0.6% SA yielded band patterns. SA protection could avoid a smear pattern associated with radiochemical reaction-induced denaturation of ^211^At-mAb. (D) Binding activity. The cellular binding activities of Sn-anti-HER2 mAb (ATE), Sn-anti-HER2 mAb (MSB), ^211^At-anti-HER2 mAb (ATE), and ^211^At-anti-HER2 mAb (MSB) were comparable to that of anti-HER2 mAb, the naked antibody. (E) Cytocidal effects. The cytotoxic effects of ^211^At-anti-HER2 mAb (ATE) and ^211^At-anti-HER2 mAb (MSB) against high HER2-expressing OE19 cells were greater than free ^211^At, whereas the cytotoxic effects of these astatinated anti-HER2 mAbs against NUGC-3 cells with low HER2 expression were comparable to that of free ^211^At. These radioactive mAbs exhibited comparable cytotoxic activities (*n* = 4). Points represent the mean, and bars represent the standard deviation.

The MSB was prepared in two steps via a PdCl_2_(PPh_3_)_2_-catalyzed reaction.^29^ We prepared trastuzumab conjugated with MSB (Sn-anti-HER2 mAb (MSB)) (Figure 1A),^5^ and determined the number of trimethylstannyls per antibody.^11^ The MSB-immunoconjugate was stored at −80 °C until use.

Using 93.5–100 MBq of ^211^At, we labeled Sn-anti-HER2 mAb (ATE) and Sn-anti-HER2 mAb (MSB) with the radionuclide, as described previously.^11^ ^211^At-mAbs were purified in PBS containing 0.6% SA (FUJIFILM Wako Pure Chemical Corporation) to avoid ROS-induced antibody denaturation (Table 1). Based on the concentration-dependent free radical scavenging activity of SA,^18^ we determined that the SA concentration was sufficient to prevent denaturation of radioactive mAbs during the labeling procedure. Radioactivity was measured using a germanium semiconductor detector (GEM P-type or GMX N-type; ORTEC, Oak Ridge, TN, USA) at RIKEN or a Curie meter (IGC-8; HITACHI, Tokyo, Japan) at the National Cancer Center.

**Table 1:** Activity and Radiochemical Yields of ^211^At-mAb**s** ^a^

antibody	coupling reagent	sodium ascorbate (%)	activity yield (MBq)	radiochemical yield (%)
trastuzumab	ATE	0.6	39.3 ± 12.9	42.3 ± 14.1
trastuzumab	MSB	0.6	40.6 ± 7.3	44.7 ± 9.5

a Astatinated mAbs were purified in PBS containing 0.6% SA, a free radical scavenger, to prevent the radioactive mAbs from being denatured. Activity and radiochemical yields are shown as mean ± standard deviation.

### 2.6 Radiochemical Yield and Radiochemical Purity

The activity yield indicated the radioactivity of the purified ^211^At-mAb, and the radiochemical yield was calculated by dividing the decay-corrected radioactivity of the purified ^211^At-mAb by the applied radioactivity. The radiochemical purity and sequential changes in purity were determined using an ultrafiltration-based analysis with minor modifications.^11^ Briefly, after purifying ^211^At-mAbs and at 9 h after the procedure, 50 kBq of ^211^At-mAbs in PBS containing 0.6% SA (final volume: 500 μL) was applied to an Amicon Ultra centrifugal filter unit with a 30K molecular weight cutoff (Merck KGaA, Darmstadt, Germany) and centrifuged for 20 min at 14,000 *g*. As a control, 50 kBq of free ^211^At (not labeled to antibodies) was processed under the same conditions. At 24 and 48 h after ^211^At-mAb preparation, 5 kBq of ^211^At-mAbs or free ^211^At was subjected to the same procedure to assess radiochemical purity over time. The applied radioactivity (50 or 5 kBq) and the radioactivity in the flow-through were measured using a gamma counter (2480 Wizard;^2^ PerkinElmer, Waltham, MA, USA). Radiochemical purity was calculated using the following formula

$$radiochemical\;purity=\left({CPM}_{50kBq}-{CPM}_{flow-through}/{FT}_{At-211}\right)/{CPM}_{50kBq}\times 100\;\text{or}\left({CPM}_{5kBq}-{CPM}_{flow-through}/{FT}_{At-211}\right)/{CPM}_{5kBq}\times 100$$

where CPM is counts per minute, and FT_At‑211_ is the flow-through correction factor calculated by dividing the CPM of the flow-through from 50 or 5 kBq of free ^211^At by the CPM of the corresponding free ^211^At applied to the filter unit.

### 2.7 Sodium Dodecyl Sulfate-Polyacrylamide Gel Electrophoresis

Following a previously established sodium dodecyl sulfate-polyacrylamide gel electrophoresis (SDS-PAGE) analysis,^11^ we evaluated the radiolysis-induced antibody denaturation.

### 2.8 Binding Activity

We evaluated the cellular binding activities of Sn-anti-HER2 mAb (ATE), Sn-anti-HER2 mAb (MSB), Sn-anti-HER2 mAb (ATE) labeled with ^211^At (^211^At-anti-HER2 mAb (ATE)), and Sn-anti-HER2 mAb (MSB) labeled with ^211^At (^211^At-anti-HER2 mAb (MSB)) using flow cytometry.^11^ The binding activities were compared with those of naked mAbs.

### 2.9 Cytocidal Effect

Using the Cell Counting Kit-8 (Dojindo, Kumamoto, Japan), we determined the cytotoxic effects of free ^211^At and astatinated mAbs on cancer cells.^11^

### 2.10 Ex Vivo Biodistribution

Mice bearing subcutaneous OE19 tumors with high HER2 expression were randomly divided into two groups and intravenously administered 1 MBq of ^211^At-anti-HER2 mAb (ATE) or ^211^At-anti-HER2 mAb (MSB). At 3.5 and 18 h after administration, blood was collected via cardiocentesis from the anesthetized mice. Subsequently, the mice were euthanized, and the tumors and normal organs were excised. After collecting the stomach contents, the stomach was washed with saline. Feces and intestinal contents were removed from the intestinal lumens, and both the small and large intestines were washed thoroughly with saline. The radioactivity of all samples was measured using a gamma counter (PerkinElmer), and the percentage of injected dose per gram of tissue (%ID/g) was calculated. For the stomach contents, we also calculated the percentage of the injected dose (%ID) to account for variations in the weight of stomach contents among mice due to differences in ingestion.

To evaluate the effect of NIS blockade on the biodistribution of ^211^At-mAb, OE19 xenograft model mice were intraperitoneally administered PBS or 1.2 μmol/g SP (Sigma-Aldrich, St. Louis, MO, USA) dissolved in PBS for 1 and 24 h before administration with 1 MBq of ^211^At-anti-HER2 mAb (ATE). Similarly, blood, normal organs, and tumors were collected 3.5 and 18 h after the injection of astatinated mAb.

### 2.11 SPECT/CT

OE19 mice were pretreated intraperitoneally with PBS or SP and administered 1 MBq ^211^At-anti-HER2 mAb (ATE). At 3.5 h after injection of the astatinated mAb, the mice were anesthetized with isoflurane and imaged using a small animal SPECT/CT scanner (NanoSPECT/CT; Mediso, Budapest, Hungary). The energy window was set at 83 keV ± 10% to detect 77–92 keV characteristic X-rays emitted through ^211^At decay.^3^

### 2.12 ^211^At-RIT under NIS Blockade

When the OE19 tumor volume reached approximately 160 mm^3^, the mice were randomly divided into four groups and administered PBS plus 0.6% SA in PBS, SP plus 0.6% SA in PBS, PBS plus 1 MBq ^211^At-anti-HER2 mAb (ATE), or SP plus 1 MBq ^211^At-anti-HER2 mAb (ATE). The mice were intraperitoneally administered PBS or 1.2 μmol/g SP at 1 and 24 h before intravenous injection of 0.6% SA in PBS or 1 MBq of ^211^At-anti-HER2 mAb (ATE) eluted in PBS containing 0.6% SA. Tumor volume and body weight were measured every 2 days. The duration of body weight loss was defined as the period between when the mice were initially administered PBS or SP (day 0) and when the lowest body weight was observed.

### 2.13 Toxicity Test

Similar to ex vivo biodistribution and SPECT/CT studies, here, mice bearing OE19 subcutaneous tumors were administered 1 MBq of ^211^At-anti-HER2 mAb (ATE) after pretreatment with PBS or SP. Blood was collected via cardiocentesis from the mice under anesthesia with isoflurane before treatment and at 1, 3, 7, and 10 days after ^211^At-RIT. The samples were immediately divided into two tubes each. One sample was used for a complete blood count, and the other was centrifuged to harvest the plasma for biochemical analysis. A Celltac α (NIHON KOHDEN, Tokyo, Japan) and DRI-CHEM NX700 (FUJIFILM, Tokyo, Japan) were used for complete blood count and biochemical analysis, respectively.

### 2.14 DSB Formation and NIS Expression in Stomach and Thyroid

OE19 model mice were administered 1 MBq of ^211^At-anti-HER2 mAb (ATE) with or without SP pretreatment. The mice were euthanized 3.5 h after ^211^At-RIT, and their stomachs and thyroids were excised. The organs were frozen in Tissue-Tek optimal cutting temperature (OCT) compound (Sakura Finetek, Tokyo, Japan) and stored at −80 °C until use.

Frozen sections (10 μm thick) were fixed with 10% formalin neutral buffer solution (Muto Pure Chemicals, Tokyo, Japan) for 10 min at room temperature (RT) and washed with PBS. The tissue sections were blocked with 5% skim milk in PBS for 1 h at RT and then incubated with a rabbit anti-γH2AX antibody (Cell Signaling Technology, Danvers, MA, USA) diluted 1:400 in the blocking buffer for 1 h at RT. Subsequently, the sections were washed with PBS and incubated with 5 μg/mL Alexa Fluor Plus 647-labeled donkey polyclonal anti-rabbit immunoglobulin antibody (Thermo Fisher Scientific) for 1 h at RT. After washing with PBS, the nuclei were stained with DAPI for 5 min at RT. Images were acquired using a fluorescence microscope (BZ-X800; Keyence, Osaka, Japan) and analyzed using the BZ-X800 Analyzer software (Keyence). Relative DSB accumulation was calculated by dividing the mean intensity of red-stained γH2AX signals in the blue-stained nucleus area in the stomach and thyroid tissues collected from mice after ^211^At-RIT by the average γH2AX intensity in each nucleus area in the corresponding tissues from nontreated mice. The thyroid region of interest was manually extracted based on follicular structure. Stomach and thyroid cells were analyzed in three and two fields of view per tissue section, respectively. To visualize NIS, tissue sections were fixed and blocked as described above and stained with a rabbit anti-NIS antibody (Osenses, Keswick, Australia) diluted 1:200 in blocking buffer for 1 h at RT. The sections were then washed with PBS and stained with 5 μg/mL Alexa Fluor Plus 647-labeled donkey polyclonal anti-rabbit immunoglobulin antibody (Thermo Fisher Scientific) and DAPI. Images were acquired using a BZ-X800 fluorescence microscope (Keyence). The expression of γH2AX and NIS was evaluated in consecutive sections.

### 2.15 Histological Examination

Mice bearing OE19 subcutaneous tumors were administered 1 MBq ^211^At-anti-HER2 mAb (ATE) after pretreatment with either PBS or SP. Before the treatment (non-treatment group), as well as at 35 days after ^211^At-RIT, the mice were euthanized. The normal organs were excised and fixed with 10% formalin neutral buffer solution (Muto Pure Chemicals) overnight at 4 °C and embedded in paraffin (Sakura Finetek). Tissue sections (3 μm thick) were visualized using hematoxylin and eosin staining.

### 2.16 Statistical Analysis

A repeated-measures analysis of variance (ANOVA) was used to analyze the sequential changes in radiochemical purity. In the ex vivo biodistribution studies, data following a normal distribution were analyzed using the Student’s *t*-test, and data that did not follow a normal distribution were analyzed using the Mann–Whitney *U* test. A repeated-measures ANOVA followed by Tukey’s post-hoc test was used to analyze tumor volume and body weight after ^211^At-RIT. The duration of body weight loss was analyzed using the Mann–Whitney *U* test. Two-way ANOVA was used to analyze the data from the toxicity study. Relative DSB accumulation was analyzed using the Mann–Whitney *U* test. Statistical analyses were performed using SPSS software version 29 (IBM, Armonk, NY, USA). Statistical significance was set at *P* < 0.05.

## 3 Results

### 3.1 ^211^At-anti-HER2 mAbs

The number of trimethylstannyl molecules per antibody in Sn-anti-HER2 mAbs (MSB) was 1.87. The activity yields of ^211^At-anti-HER2 mAb (ATE) and ^211^At-anti-HER2 mAb (MSB) were 39.3 ± 12.9 MBq and 40.6 ± 7.3 MBq, respectively (Table 1). Radiochemical yield of ^211^At-anti-HER2 mAb (ATE) and ^211^At-anti-HER2 mAb (MSB) was 42.3 ± 14.1% and 44.7 ± 9.5%, respectively (Table 1). The radiochemical purities of ^211^At-anti-HER2 mAb (ATE) and ^211^At-anti-HER2 mAb (MSB) were 98% and 97%, respectively (Figure 1B). Although gradual ^211^At release was observed for both radioactive mAbs, the amount of free radionuclides in the ^211^At-anti-HER2 mAb (ATE) solution was significantly lower than that in the ^211^At-anti-HER2 mAb (MSB) solution (*P* < 0.001) (Figure 1B). The decrease in radiochemical purity reached a plateau 24 h after purification for astatinated mAbs. The purities of ^211^At-anti-HER2 mAb (ATE) and ^211^At-anti-HER2 mAb (MSB) were approximately 92% and 86%, respectively.

### 3.2 SA Protection to Avoid ^211^At-Induced Antibody Denaturation

In SDS-PAGE analysis, ^211^At-anti-HER2 mAbs eluted in PBS containing 0.6% SA were observed as band patterns, similar to those of the corresponding Sn-anti-HER2 mAbs (Figure 1C). SA in the elution buffer prevented a smear pattern associated with radiochemical reaction-induced denaturation of ^211^At-mAb.^5,11,18^ Accordingly, flow cytometry analysis showed that the cellular binding activity of ^211^At-anti-HER2 mAbs was comparable to that of Sn-anti-HER2 mAbs and anti-HER2 mAb, the naked antibody, suggesting that SA protection successfully prevented the disturbance in binding activity caused by ^211^At-induced antibody denaturation (Figure 1D).

### 3.3 Cytocidal Effect

Both ^211^At-anti-HER2 mAb (ATE) and ^211^At-anti-HER2 mAb (MSB) exerted comparable cytocidal effects, depending on HER2 expression levels (Figure 1E). The killing activity against OE19 cells with high HER2 expression was greater than ^211^At, whereas the activity against NUGC-3 cells expressing low HER2 was comparable to that of ^211^At.

### 3.4 Comparison of RIT Biodistribution between ^211^At-anti-HER2 mAb (ATE) and ^211^At-anti-HER2 mAb (MSB)

Both ^211^At-anti-HER2 mAb (ATE) and ^211^At-anti-HER2 mAb (MSB) accumulated in the tumor compared to normal organs, whereas no difference was observed in tumor accumulation and distribution in normal organs between these radioactive mAbs (Figure 2A). The %ID and %ID/g for the stomach contents at 3.5 h after administration of ^211^At-anti-HER2 mAb (MSB) tended to be higher than those after administration of ^211^At-anti-HER2 mAb (ATE); however, the differences were not significant (%ID, *P* = 0.091; %ID/g, *P* = 0.222) (Figure 2A,B). At 18 h after administration, the %ID for the stomach contents in the ^211^At-anti-HER2 mAb (ATE) group was significantly higher than in the ^211^At-anti-HER2 mAb (MSB) group, accompanied by a nonsignificant trend toward higher %ID/g (%ID, *P* = 0.042; %ID/g, *P* = 0.442) (Figure 2A,B).

**Figure 2:**
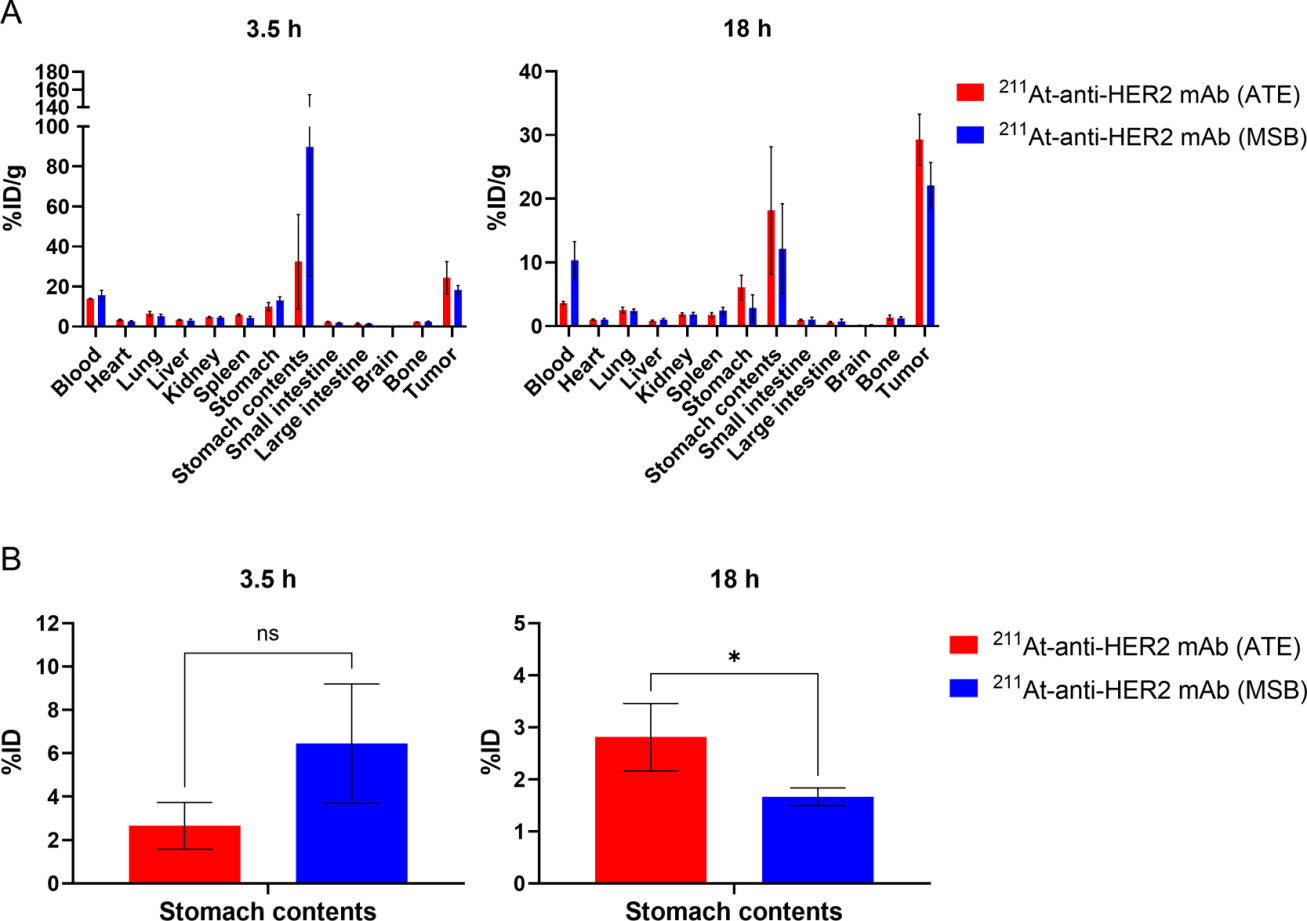
Ex vivo biodistribution study after administration of ^211^At-anti-HER2 mAb (ATE) or ^211^At-anti-HER2 mAb (MSB). (A) %ID/g for the tumor, normal organs, and stomach contents. Mice bearing the OE19 tumors were administered 1 MBq of ^211^At-anti-HER2 mAb (ATE) or ^211^At-anti-HER2 mAb (MSB). At 3.5 and 18 h after injection, tumors and normal organs were collected, and %ID/g was calculated. Data are shown as mean ± standard deviation. Three mice were examined in each group. (B) %ID for stomach contents. Data are shown as mean ± standard deviation. Three mice were examined in each group. *, *P* < 0.05.

### 3.5 Altered Biodistribution via Competitive Inhibition of NIS with SP

The competitive inhibition of NIS with SP significantly reduced the %ID/g for the stomach and stomach contents at 3.5 h after the administration of ^211^At-anti-HER2 mAb (ATE) (stomach, *P* = 0.020; stomach contents, *P* = 0.013) (Figure 3A). In contrast, at 18 h after ^211^At-RIT, no significant difference was observed in the %ID/g for the stomach and stomach contents between the groups treated with PBS plus ^211^At-anti-HER2 mAb (ATE) and SP plus ^211^At-anti-HER2 mAb (ATE) (stomach, *P* = 0.103; stomach contents, *P* = 0.204) (Figure 3A). The %ID for the stomach contents at 3.5 and 18 h after administration of SP plus ^211^At-anti-HER2 mAb (ATE) group showed a lower tendency than that in the PBS plus ^211^At-anti-HER2 mAb (ATE) group; however, the differences were not statistically significant (3.5 h, *P* = 0.218; 18 h, *P* = 0.059) (Figure 3B). Regarding the large intestine 3.5 h after administration of the radioactive mAb and the spleen 18 h after injection, NIS inhibition resulted in a significantly higher %ID/g (large intestine, *P* = 0.020; spleen, *P* = 0.003) (Figure 3A). There was no difference in %ID/g for tumors at 3.5 and 18 h after the administration of the astatinated mAb between the groups treated with PBS plus ^211^At-anti-HER2 mAb (ATE) and SP plus ^211^At-anti-HER2 mAb (ATE) (3.5 h, *P* = 0.688; 18 h, *P* = 0.838) (Figure 3A).

**Figure 3:**
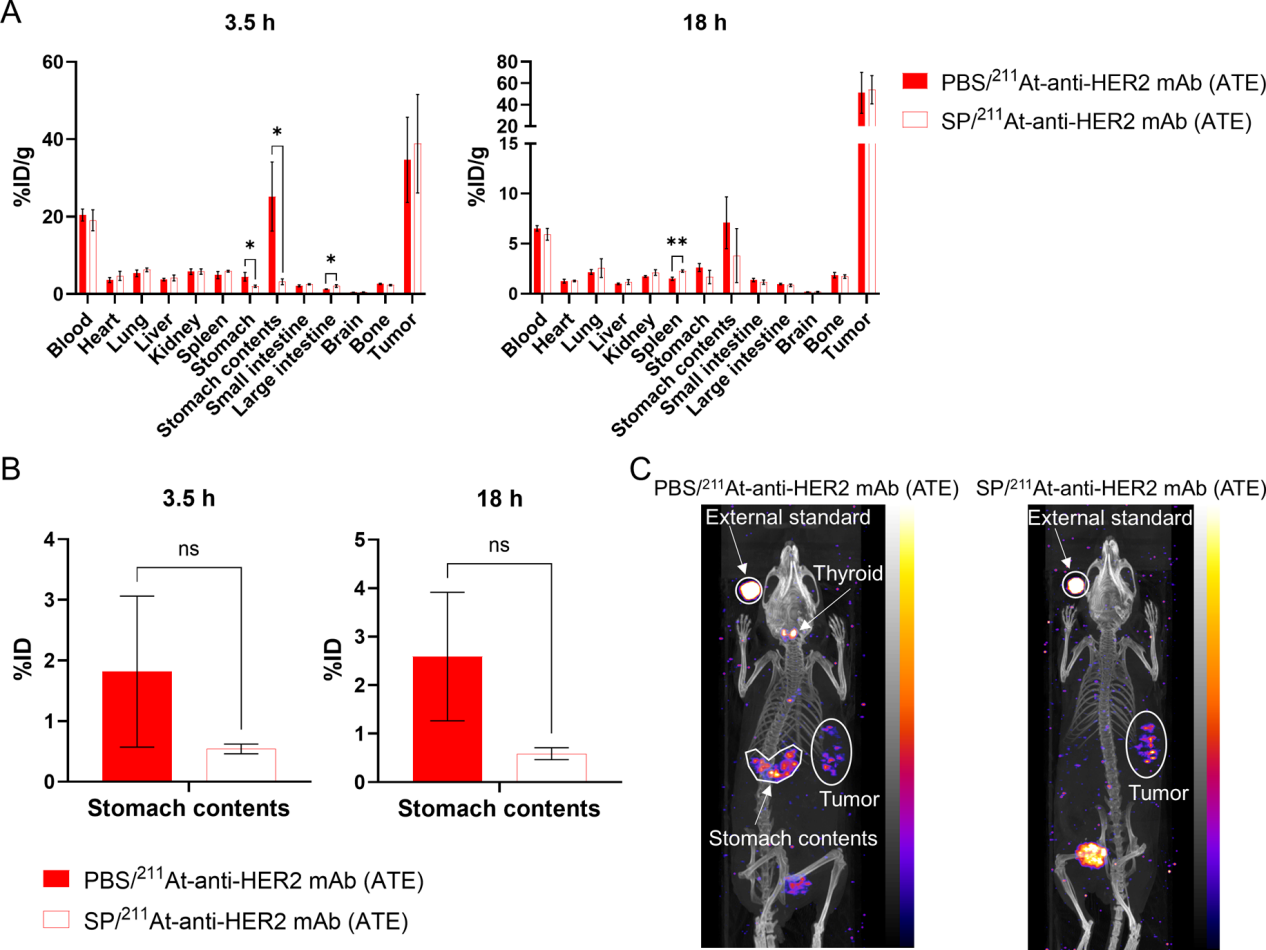
Altered biodistribution via pretreatment with SP, an NIS inhibitor, in ^211^At-RIT. (A) Ex vivo biodistribution study. After pretreatment with PBS or SP, mice bearing OE19 tumors were administered 1 MBq of ^211^At-anti-HER2 mAb (ATE). At 3.5 and 18 h after the administration of ^211^At-mAb, tumors and normal organs were collected, and %ID/g was calculated. Data are shown as mean ± standard deviation. Three mice were examined in each group. *, *P* < 0.05; **, *P* < 0.005. (B) %ID for the stomach contents. Data are presented as mean ± standard deviation. Three mice were examined in each group. (C) SPECT/CT. Model mice were administered 1 MBq of ^211^At-anti-HER2 mAb (ATE) after pretreatment with PBS or SP, and SPECT/CT images were acquired 3.5 h after administration of the radioactive mAb. The radioactivity of the external standard was one-tenth of the administered radioactivity.

### 3.6 SPECT/CT

In the OE19 xenograft model administered ^211^At-anti-HER2 mAb (ATE) after pretreatment with PBS, signals in the thyroid and stomach contents were observed (Figure 3C). In contrast, pretreatment with SP reduced the signals in the thyroid and stomach contents, which were difficult to visualize (Figure 3C).

### 3.7 ^211^At-RIT under Competitive Inhibition of NIS

Both PBS plus ^211^At-anti-HER2 mAb (ATE) and SP plus ^211^At-anti-HER2 mAb (ATE) significantly reduced tumor growth (PBS/0.6% SA vs PBS/^211^At-anti-HER2 mAb (ATE), *P* < 0.001; SP/0.6% SA vs SP/^211^At-anti-HER2 mAb (ATE), *P* < 0.001), whereas their antitumor activities were comparable (*P* = 0.903) (Figure 4A).

**Figure 4:**
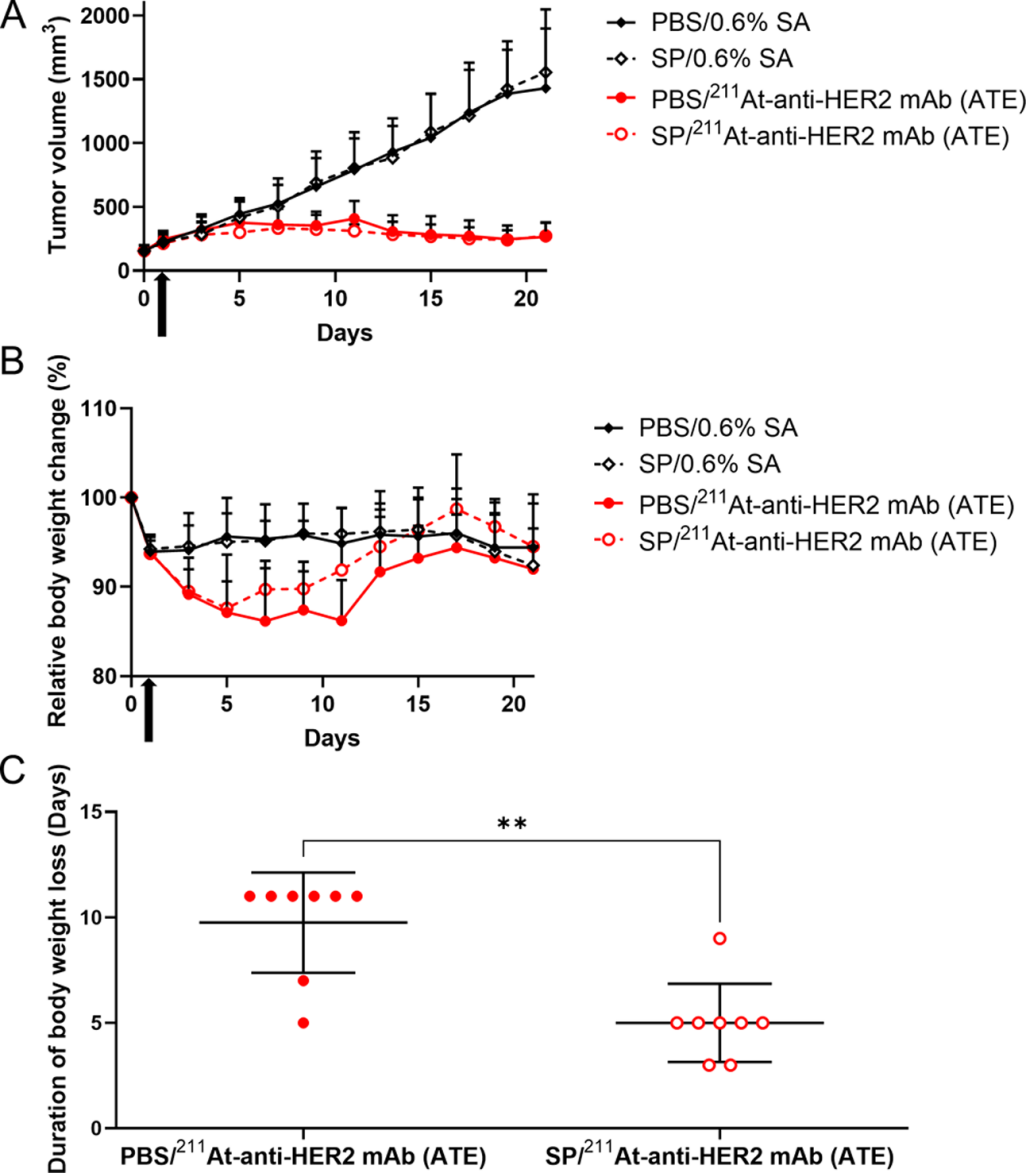
^211^At-RIT under competitive inhibition of NIS. (A) Antitumor activity. Mice bearing OE19 tumors were administered 1 MBq of ^211^At-anti-HER2 mAb (ATE) after pretreatment with PBS or SP. Arrows, ^211^At-anti-HER2 mAb (ATE) administration; points, mean; bars, standard deviation; number of animals per group, *n* = 8. (B) Body weight loss. Similarly, the model mice were treated with astatinated mAb. Arrows, ^211^At-anti-HER2 mAb (ATE) administration; points, mean; bars, standard deviation; number of animals per group, *n* = 8. (C) Duration of body weight loss. This was defined as the period between when mice were initially administered PBS or SP (day 0) and when the lowest body weight was observed. Data are shown as mean ± standard deviation. Points, duration of body weight loss, and number of animals per group (*n* = 8). **, *P* < 0.005.

Although significant body weight loss was observed after ^211^At-RIT (PBS/0.6% SA vs PBS/^211^At-anti-HER2 mAb (ATE), *P* < 0.001; SP/0.6% SA vs SP/^211^At-anti-HER2 mAb (ATE), *P* = 0.009), NIS inhibition with SP resulted in significantly milder body weight loss (PBS/^211^At-anti-HER2 mAb (ATE) vs SP/^211^At-anti-HER2 mAb (ATE), *P* < 0.001) (Figure 4B). Moreover, the duration of body weight loss under competitive inhibition of NIS by SP was significantly shorter than that without pretreatment (*P* = 0.002) (Figure 4C).

### 3.8 Toxicity Test

Leukopenia and thrombocytopenia were observed after RIT with 1 MBq of ^211^At-anti-HER2 mAb (ATE) and reached a nadir at 3 and 7 days after administration, respectively. There was no difference in leukopenia or thrombocytopenia between the PBS/^211^At-anti-HER2 mAb (ATE) and SP/^211^At-anti-HER2 mAb (ATE) groups (white blood cell (WBC) count, *P* = 0.676; platelet (PLT) count, *P* = 0.498) (Figure 5). Similarly, hemoglobin (Hb) levels were comparable between the groups (*P* = 0.270) (Figure 5). Although the red blood cell (RBC) count in the SP/^211^At-anti-HER2 mAb (ATE) group was significantly lower than that in the PBS/^211^At-anti-HER2 mAb (ATE) group (*P* = 0.028) (Figure 5), ^211^At-RIT under the NIS inhibition with SP did not cause a significant decrease in RBC count and Hb value (RBC, *P* = 0.257; Hb, *P* = 0.142) (Figure S1). After ^211^At-RIT, transient hepatotoxicity was observed, whereas no difference was observed in liver damage between the PBS/^211^At-anti-HER2 mAb (ATE) and SP/^211^At-anti-HER2 mAb (ATE) groups (aspartate aminotransferase (AST), *P* = 0.523; alanine aminotransferase (ALT), *P* = 0.089; lactate dehydrogenase (LDH), *P* = 0.525) (Figure 5). RIT with 1 MBq of ^211^At-anti-HER2 mAb (ATE) did not cause renal toxicity, and there was no difference in blood urea nitrogen (BUN) or creatinine (Cre) levels between the groups (BUN, *P* = 0.312; Cre, *P* = 0.294) (Figure 5).

**Figure 5:**
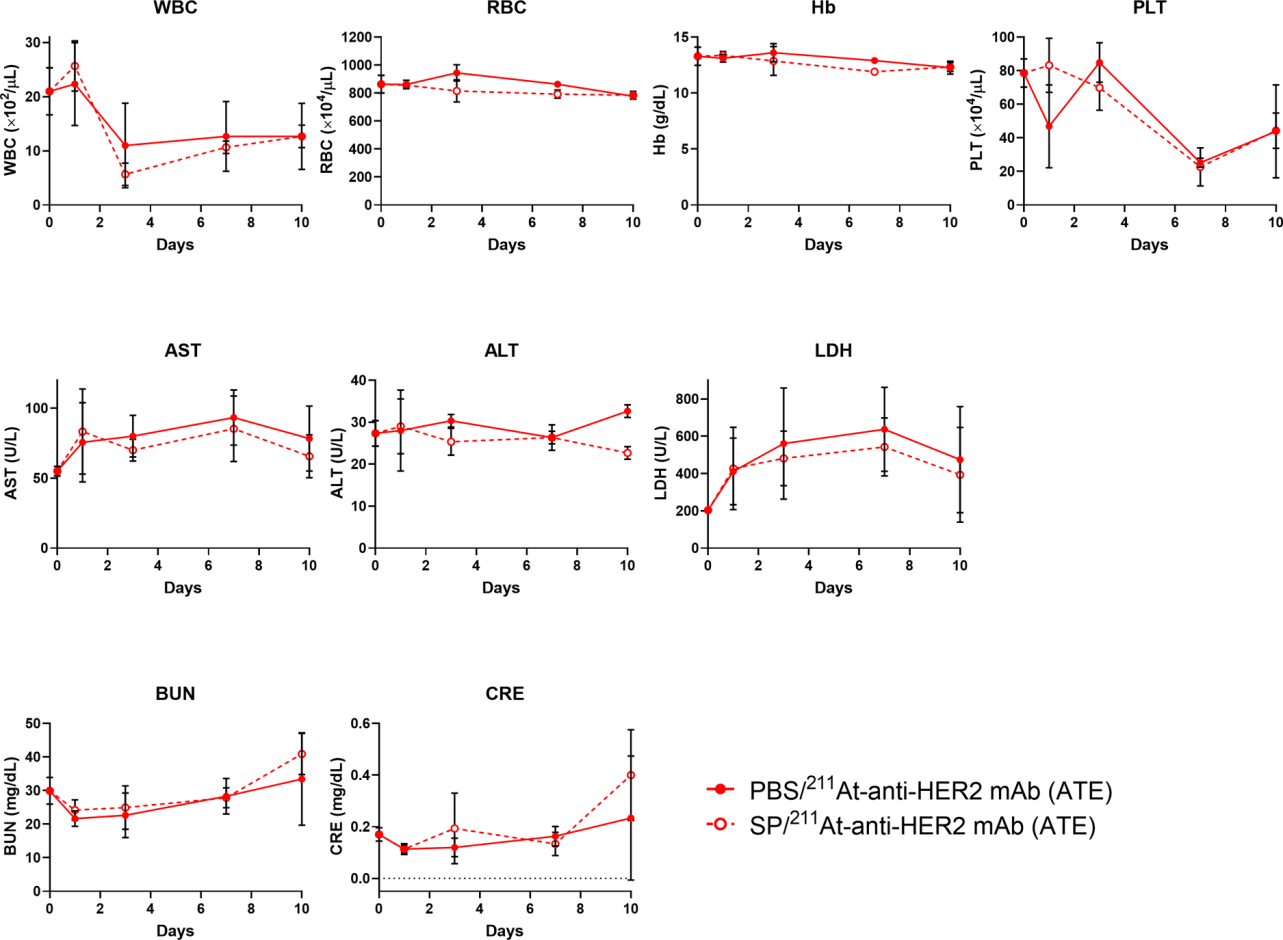
Toxicity test. Mice bearing OE19 tumors were administered 1 MBq of ^211^At-anti-HER2 mAb (ATE) after pretreatment with PBS or SP. Before the treatment (day 0), as well as at 1, 3, 7, and 10 days after ^211^At-RIT, blood was collected, and hematotoxicity, hepatotoxicity, and renal toxicity were evaluated. Points, mean; bars, standard deviation; number of animals per group, *n* = 3.

### 3.9 DSB Formation in Stomach and Thyroid

After RIT with 1 MBq ^211^At-anti-HER2 mAb (ATE), DSBs were formed heterogeneously in the stomach. In contrast, homogeneous DSB formation was observed in the thyroid tissue (Figure 6A). NIS expression in the stomach was relatively high in cells near the gastric lumen (Figure 6A), whereas thyroid cells homogeneously express NIS (Figure 6A). In the stomach, DSBs accumulated in the cells expressing relatively high levels of NIS (Figure 6A). Inhibition of NIS with SP significantly reduced DSB formation in the stomach and thyroid (stomach, *P* < 0.001; thyroid, *P* < 0.001) (Figure 6B).

**Figure 6:**
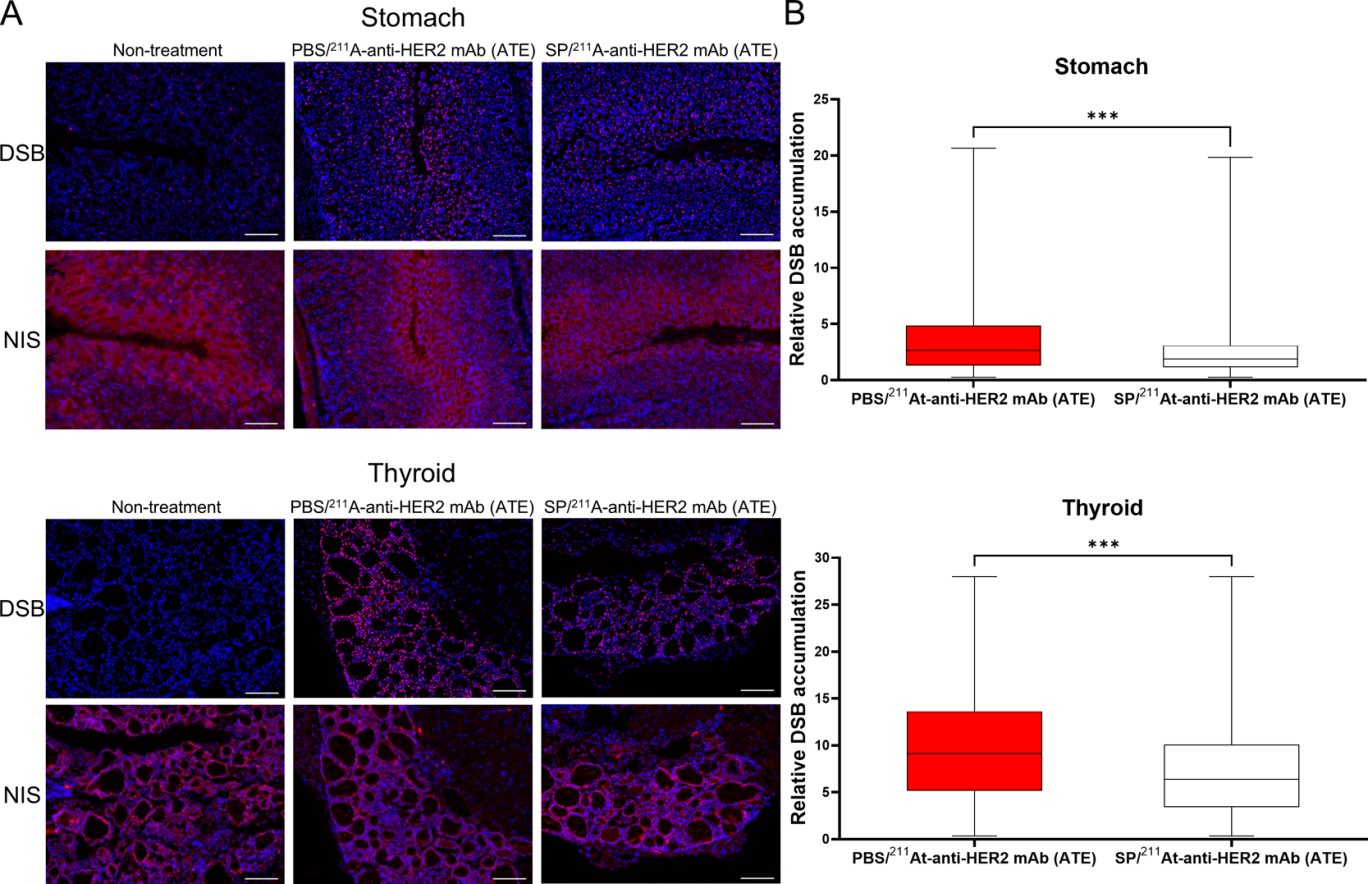
DSB formation in the stomach and thyroid after ^211^At-RIT. (A) Fluorescent immunohistostaining. The nucleus is indicated in blue, and γH2AX and NIS are indicated in red. At 3.5 h after RIT with 1 MBq of ^211^At-anti-HER2 mAb (ATE), γH2AX and NIS expressions were evaluated on consecutive sections. Scale bar: 100 μm. (B) Box and whisker plots showing relative DSB accumulation in the stomach and thyroid following ^211^At-RIT. Three mice were examined in each group. Number of counted cells: stomach in PBS/^211^At-anti-HER2 mAb (ATE) group, 21,668 cells; stomach in SP/^211^At-anti-HER2 mAb (ATE) group, 21,229 cells; thyroid in PBS/^211^At-anti-HER2 mAb (ATE) group, 6,533 cells; thyroid in SP/^211^At-anti-HER2 mAb (ATE) group, 4,628 cells. ***, *P* < 0.001.

### 3.10 Histological Findings in Stomach and Thyroid

The stomach and thyroid were histologically examined 35 days after the administration of 1 MBq of ^211^At-anti-HER2 mAb (ATE). There was no difference in the stomach findings between the groups pretreated with PBS and SP (Figure 7). The stomach findings after ^211^Ar-RIT were similar to those in the non-treatment group (Figure 7). In contrast, the follicular structure in the thyroid was disturbed in one of the three model mice pretreated with PBS (Figure 7; mouse 1 in the PBS/^211^At-anti-HER2 mAb group). In the group pretreated with SP, NIS inhibition maintained thyroid follicles in all model mice after ^211^At-RIT (Figure 7).

**Figure 7:**
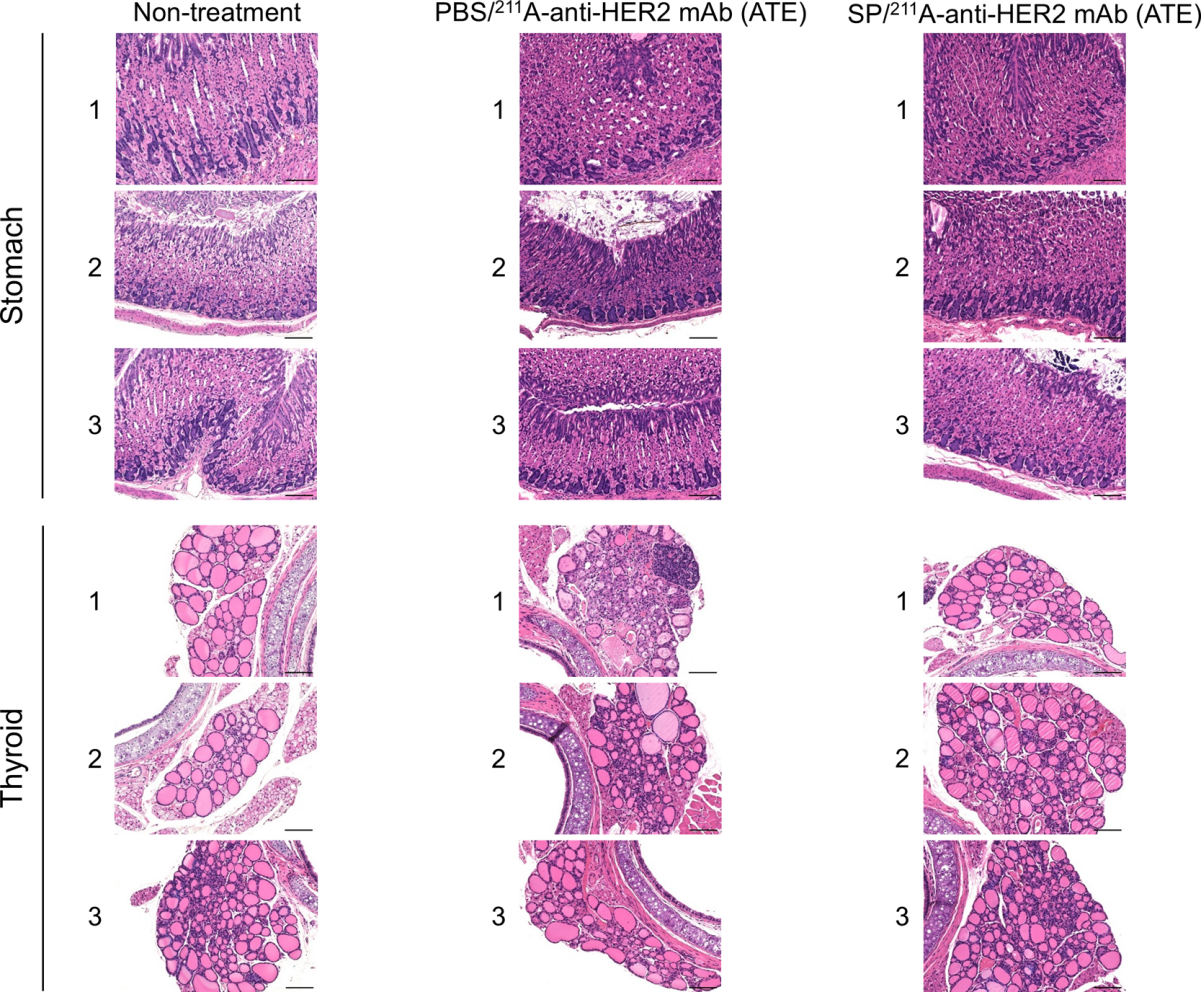
Histological findings of the stomach and thyroid 35 days after ^211^At-RIT. The stomach and thyroid were visualized using hematoxylin and eosin staining. There was no difference in the stomach findings between the non-treatment, PBS/^211^At-anti-HER2 mAb (ATE), and SP/^211^At-anti-HER2 mAb (ATE) groups. The thyroid follicular structure was distributed in one of the three model mice pretreated with PBS (mouse 1 in the PBS/^211^At-anti-HER2 mAb group), whereas the follicles were maintained in all model mice pretreated with SP. Three mice were examined in each group. Scale bar: 100 μm.

## 4 Discussion

Regarding the activity yield (Table 1), radiochemical yield (Table 1), binding activity (Figure 1D), and cytocidal activity (Figure 1E), ^211^At-anti-HER2 mAb (ATE) and ^211^At-anti-HER2 mAb (MSB) were comparable. Immunoglobulin G1 (IgG1) has approximately 40 modifiable lysine residues on its surface.^31^ ATE molecules are stochastically conjugated, and controlling the conjugation site is impossible.^32^ Although it was considered that ATE molecules attached to lysine residues in or near the antigen-binding site through the random conjugation process might disturb the binding activity of trastuzumab,^32^ the attachment of the coupling reagent to the antibody did not significantly impact the cellular binding activity (Figure 1D). This finding is consistent with that of a previous study.^20^

SDS-PAGE analysis indicated that ATE or MSB conjugation, as well as ^211^At labeling, may cause antibody fragmentation, including the generation of fragments with molecular weights of approximately 25 kDa (Figure 1C). Although we did not quantify the proportion of these fragments in ^211^At-mAb solutions—a limitation in our study—the fragmented antibodies, such as fragment antigen-binding (Fab), appear to represent only a minor fraction. First, in the SDS-PAGE visualized with silver staining, fragments with a lower molecular weight than 100 kDa were barely detectable. In contrast, their visualization by autoradiography suggests heterogeneous ^211^At-labeling across the antibody molecules. Second, in the biodistribution study (Figure 2A), the %ID/g in the kidney was clearly lower than that reported for radioactive Fab or F­(ab’)_2_ trastuzumab, which are predominantly cleared renally,^33^ indicating Fab and F­(ab′)_2_ represent only a small population in our ^211^At-mAb preparations. In this study, radiochemical purity was determined using an ultrafiltration-based method. Because the purity obtained by this procedure was consistent with that measured by protein precipitation using methanol,^5^ it is reasonable to conclude that this procedure adequately distinguished radioactive immunoconjugates from free ^211^At released from antibodies. However, because we used an Amicon Ultra centrifugal filter unit with a molecular weight cutoff of 30K, the radiochemical purity must be interpreted with caution, as astatinated light chains passing through the filter into the flow-through could have an influence on the measured purity. Aneheim et al. reported that astatinated MSB immunoconjugates exhibited minimal ^211^At release in PBS containing human serum albumin (HSA).^20^ In contrast, in our ^211^At-anti-HER2 mAb (MSB) solution purified in PBS containing 0.6% SA, gradual ^211^At release was observed (Figure 1B). In addition to the type of storage solutions, the stability of ^211^At-labeled immunoconjugates containing a thiosuccinimide linkage appears to depend on the storage environment—including temperature—as well as the nature of the tumor targeting carrier (full-length antibody, nanobody, or single-domain antibody (sdAb)). Using *N*-succinimidyl 4-(1,2-bis-*tert*-butoxycarbonyl)­guanidinomethyl-3-(trimethylstannyl)­benzoate, ATE, or MSB, Dekempeneer et al. labeled anti-HER2 nanobody with ^211^At and evaluated the radiochemical purity in PBS at RT, PBS at 37 °C, and serum at 37 °C for 24 h.^34^ Gradual deastatination was observed only in MSB-conjugated nanobodies incubated in PBS at 37 °C, whereas this was not clearly seen in the other conjugates.^34^ Feng et al. prepared anti-HER2 sdAb conjugates using thiol-selective coupling reagents containing either a maleimide moiety or a phenyloxadiazolyl methylsulfone (PODS) moiety, and labeled these immunoconjugates with ^211^At.^35^ The comparison of stability in PBS, 50 mM cysteine, and HSA-containing PBS revealed that PODS-based astatinated sdAb successfully avoided the degradation observed in maleimide-based radioactive sdAb in cysteine and HSA solutions.^35^ In our study, ^211^At release was also observed in the ^211^At-anti-HER2 mAb (ATE) solution. However, radionuclide release was significantly lower than that observed in the ^211^At-anti-HER2 mAb (MSB) solution (*P* < 0.001) (Figure 1B). Consistent with these findings, Dekempeneer et al. also reported that astatinated ATE-conjugated nanobodies exhibited less deastatination than radioactive MSB-conjugated nanobodies.^34^ Free ^211^At is secreted into the gastric lumen.^19^ Therefore, the relatively abundant free ^211^At released from antibodies in the ^211^At-anti-HER2 mAb (MSB) solution may cause a higher tendency in %ID and %ID/g for stomach contents at 3.5 h after administration of ^211^At-anti-HER2 mAb (MSB) than those in the ^211^At-anti-HER2 mAb (ATE) group (%ID, *P* = 0.091; %ID/g, *P* = 0.222) (Figure 2A,B).

A direct comparison of biodistribution after the administration of ^211^At-anti-HER2 mAb (ATE) or ^211^At-anti-HER2 mAb (MSB) revealed no difference in tumor accumulation and distribution in normal organs between the groups (Figure 2A). Considering free ^211^At accumulation in the stomach content, stomach, and thyroid,^4,19^ we initially presumed that the coupling reagents, which determine ^211^At liberation in the storage solution and in vivo, led to differences in radionuclide uptake in these organs. However, ATE and MSB did not significantly affect the stomach uptake of ^211^At-RIT under SA protection (at 3.5 h, *P* = 0.117; at 18 h, *P* = 0.127) (Figure 2A). Although the %ID for the stomach contents at 18 h after administration of ^211^At-anti-HER2 mAb (ATE) was significantly higher than that after administration of ^211^At-anti-HER2 mAb (MSB) (*P* = 0.042) (Figure 2B), there was no difference in ^211^At uptake in the distal segments of the digestive tract—such as the small and large intestines—between these groups (small intestine, *P* = 0.818; large intestine, *P* = 0.553) (Figure 2A). To achieve a favorable stability and tumor-specific targeting in vivo, and to guide the selection of bioconjugation chemistry while avoiding conditions that cause deastatination—such as the hydrolysis of the thiosuccinimide linkage—a comprehensive strategy is required. This includes incorporating intracellular residualization functionalities into ^211^At-labeled carriers,^35^ and introducing substituents adjacent to the ^211^At atom in an aromatic ring to enhance the stability of astatinated aromatic compounds against deastatination.^36^

Through a direct comparison study, Aneheim et al. showed that MSB-attached MX35, a murine anti-sodium-dependent phosphate-transport protein 2b (NaPi2b) antibody, labeled with ^211^At, showed a significantly lower %ID/g in blood at 25.4 h after administration than radioactive ATE-attached MX35.^20^ In contrast, compared to ^211^At-anti-HER2 mAb (ATE), ^211^At-anti-HER2 mAb (MSB) was retained in the blood circulation 18 h after administration (Figure 2A). These studies present different findings related to the half-life within blood circulation. Thus, besides the coupling reagent that determines ^211^At liberation, the solubility of immunoconjugates^37^ and antibody characterization, such as humanized or murine antibodies, may determine the half-life of ^211^At-mAb. Evaluating metabolites in vivo can help improve our understanding of the pharmacokinetics of ^211^At-mAbs and the factors influencing antibody-based ^211^At delivery systems,^38,39^ while also providing insights for developing strategies to enhance stability and tumor specificity.

Ex vivo biodistribution study showed that in both ^211^At-anti-HER2 mAb (ATE) and ^211^At-anti-HER2 mAb (MSB) groups, a higher %ID/g in the tumor relative to the blood was observed at 18 h after administration (Figure 2A). In ^211^At RIT, compared with passive targeting based on the enhanced permeability and retention (EPR) effect,^40^ active targeting via an antigen–antibody reaction predominantly contributes to higher radioactivity accumulation in tumors than in the blood.^5^ Thus, during the processes of coupling reagent conjugation, ^211^At labeling, and storage until administration, it is pivotal to maintain the specific binding activity of radioactive antibodies to achieve successful tumor accumulation. The binding activities of Sn-anti-HER2 mAb (ATE) and Sn-anti-HER2 mAb (MSB) were comparable to those of the naked anti-HER2 mAb (Figure 1D). This finding indicates that the attachment of these coupling reagents did not affect cellular binding activity. During the process from ^211^At labeling to administration, ROS generated through the radiolysis of water induce denaturation of ^211^At-mAbs, thereby disturbing the specific binding activity.^5,11,18^ We previously demonstrated that ^211^At-induced antibody denaturation yielded a smear pattern in SDS-PAGE analysis.^5,11,18^ In contrast, SDS-PAGE showed a band pattern for both ^211^At-anti-HER2 mAb (ATE) and ^211^At-anti-HER2 mAb (MSB) (Figure 1C), suggesting that antibody denaturation was prevented. Consistent with this finding, here, the cellular binding activity of the astatinated antibodies was comparable to that of the naked antibody (Figure 1D). To quench ROS, we purified ^211^At-labeled antibodies in PBS containing 0.6% SA, a free radical scavenger. These results imply that SA in the elution buffer protected ^211^At-anti-HER2 mAb (ATE) and ^211^At-anti-HER2 mAb (MSB) from ^211^At-induced antibody denaturation and maintained the specific binding activity of the radioactive antibodies, resulting in a higher %ID/g in the tumor relative to the blood via well-maintained active targeting (Figure 2A).

To evaluate the competitive inhibition of NIS in ^211^At-RIT pharmacologically and toxicologically, we chose ATE as a coupling reagent for ^211^At labeling because the smaller sequential change in radiochemical purity can contribute to better reproducibility (Figure 1B).

Consistent with the findings of previous studies,^27,28^ pretreatment with SP, a competitive inhibitor of NIS, significantly reduced %ID/g in the stomach 3.5 h after administration of ^211^At-anti-HER2 mAb (ATE) (*P* = 0.020) (Figure 3A), as indicated by our ex vivo biodistribution study. ^211^At is secreted into gastric juice after uptake by stomach cells,^19^ which is a pharmacological characteristic of halogens, such as iodine.^21,22,25,26^ Thus, the pretreatment-induced reduction in gastric uptake reasonably explains the significant decrease in %ID/g for stomach contents at 3.5 h after administration of ^211^At-anti-HER2 mAb (ATE) (*P* = 0.013), as well as the trends toward lower %ID/g for the stomach contents at 18 h after ^211^At-RIT and lower %ID values for the contents (Figure 3A,B). Similarly, SPECT/CT images revealed that competitive inhibition of NIS reduced the signals in the thyroid 3.5 h after the administration of the radioactive antibody (Figure 3C). However, SP did not show a long duration of action because 18 h after the administration of ^211^At-anti-HER2 mAb (ATE), there was no significant difference in the %ID/g value for the stomach between the groups pretreated with PBS and SP (*P* = 0.103) (Figure 3A).

Besides thyroid and gastric mucosal cells, NIS is located on the apical membrane of the human duodenum.^22^ Similarly, in mice and rats, NIS is expressed in the duodenum, jejunum, and ileum.^41^ Previous studies have suggested that iodine secreted into gastric juice is transported via NIS in the small intestine and reabsorbed into systemic circulation.^22,41^ The transportation system via NIS in the apical membrane seems to result in the small intestine uptake of ^211^At. However, in our study, there was no difference in the %ID/g value for the small intestine between groups treated with PBS plus ^211^At-anti-HER2 mAb (ATE) and SP plus ^211^At-anti-HER2 mAb (ATE) (3.5 h, *P* = 0.074; 18 h, *P* = 0.25) (Figure 3A), and NIS inhibition with SP did not affect ^211^At uptake. In mice, the expression level of NIS messenger ribonucleic acid (mRNA) in the small intestine is lower than that in the thyroid and stomach.^23^ Owing to the relatively low NIS expression, competitive inhibition with SP may not influence ^211^At uptake in the small intestine after the administration of astatinated mAb (Figure 3A). Similarly, insufficient reabsorption activity—presumably due to the relatively low NIS expression in mice—may explain why ^211^At present in the stomach contents did not influence its uptake in the small intestine (Figures 2A,B and 3A,B).

In contrast to those in the stomach and stomach contents, pretreatment with SP resulted in significantly higher %ID/g values for the large intestine at 3.5 h after administration of ^211^At-mAb and the spleen at 18 h after injection (Figure 3A). However, higher ^211^At uptake did not cause histological changes in the large intestinal tissues, and there was no difference in the findings between the nontreated, PBS plus ^211^At-anti-HER2 mAb (ATE), and SP plus ^211^At-anti-HER2 mAb (ATE) groups (Figure S2). Regarding the spleen, although more cellular infiltration was observed in the red pulp after ^211^At-RIT than in the non-treatment group, implying an inflammatory response in the hematopoietic system after exposure to radiation,^42^ there was no significant difference in infiltration between the groups treated with PBS plus ^211^At-anti-HER2 mAb (ATE) and SP plus ^211^At-anti-HER2 mAb (ATE) (Figure S3A–C). Thus, the toxic effects of SP on ^211^At-RIT were not observed in the large intestine or spleen.

Regarding tumor accumulation, the %ID/g values for the tumors were comparable between the groups treated with PBS plus ^211^At-anti-HER2 mAb (ATE) and SP plus ^211^At-anti-HER2 mAb (ATE) (3.5 h, *P* = 0.688; 18 h, *P* = 0.838) (Figure 3A). Consistently, there was no difference in antitumor effects between these groups (*P* = 0.903) (Figure 4A). Thus, NIS inhibition by SP did not attenuate tumor accumulation or antitumor activity of ^211^At-RIT. In contrast, tumor-selective delivery of ^211^At via NIS inhibition resulted in significantly lower body weight loss (PBS/^211^At-anti-HER2 mAb (ATE) vs SP/^211^At-anti-HER2 mAb (ATE), *P* < 0.001) (Figure 4B). Moreover, mice pretreated with SP showed a more rapid recovery from body weight loss after ^211^At-RIT (*P* = 0.002) (Figure 4C). Therefore, tumor-selective delivery appears to contribute significantly to milder toxicity without attenuating the antitumor effect of ^211^At-RIT.

To evaluate the toxicity and tolerability of combining SA protection to avoid ^211^At-induced antibody denaturation and competitive inhibition of NIS with SP for tumor-selective delivery of ^211^At, we analyzed blood samples collected from mice after administration of the astatinated mAb. Under NIS inhibition with SP, ^211^At-RIT did not significantly decrease the RBC count or Hb value (RBC, *P* = 0.257; Hb, *P* = 0.142) (Figure S1). Transient leukopenia, thrombocytopenia, and hepatic damage were observed, and these toxicities were comparable between the groups treated with PBS plus ^211^At-anti-HER2 mAb (ATE) and SP plus ^211^At-anti-HER2 mAb (ATE) (WBC, *P* = 0.676; PLT, *P* = 0.498; AST, *P* = 0.523; ALT, *P* = 0.089; LDH, *P* = 0.525) (Figure 5). No renal toxicity was observed after ^211^At-RIT (Figure 5). Therefore, it is feasible to add competitive inhibition of NIS by SP to ^211^At-RIT under SA protection.

In stomach tissues, NIS expression was heterogeneous and relatively high in cells near the gastric lumen (Figure 6A), consistent with immunohistological findings showing that NIS is expressed in the gastric mucosal cells.^21,22,24^ In contrast, NIS was homogeneously expressed in thyroid follicular cells (Figure 6A). After ^211^At-RIT, DSB accumulated in stomach cells adjacent to the gastric lumen, whereas homogeneous DSB formation was observed in thyroid follicular cells (Figure 6A). Considering these findings in consecutive tissue sections, ^211^At-induced DSB formation in the stomach and thyroid appears to depend on NIS expression levels. Competitive inhibition of NIS with SP significantly reduced DSB accumulation in these tissues (stomach, *P* < 0.001; thyroid, *P* < 0.001) (Figure 6B). At 35 days after ^211^At-RIT, histological analysis did not show ^211^At-induced damage in the stomach tissues in either the group administered PBS plus ^211^At-anti-HER2 mAb (ATE) or SP plus ^211^At-anti-HER2 mAb (ATE) (Figure 7). Thus, DSB accumulation in the stomach after ^211^At-RIT did not cause irreversible histological alterations (Figures 6A,B and 7). The high regenerative activity of gastric mucosal cells may contribute to this histological finding.^43–46^ In contrast, the follicular structure in the thyroid was disturbed in one out of three model mice pretreated with PBS (Figure 7; mouse 1 in the PBS/^211^At-anti-HER2 mAb group), whereas competitive inhibition of NIS with SP successfully maintained the thyroid follicles in all model mice after administration of ^211^At-mAb (Figure 7). These histological findings suggest that in ^211^At-RIT, NIS inhibition is reasonable in terms of maintaining the histological structure of the thyroid.

Compared with the histological findings, such as the disappearance of follicles and fibrosis at 29 and 60 days after administration of 1 MBq of [^211^At]­NaAt,^19^ which accumulates in the stomach and thyroid,^4,19^ the structural changes in the thyroid after administration of PBS plus 1 MBq of ^211^At-mAb were milder (Figure 7). In ^211^At-RIT, even if NIS inhibition is not used, an antibody-based delivery system for tumor-selective ^211^At accumulation must reduce the radiation dose to the thyroid compared to [^211^At]­NaAt administration, resulting in milder histological changes.

Attenuated ^211^At distribution in the stomach and thyroid after ^211^At-RIT with NIS inhibition (Figure 3A,C), resulting in significantly less DNA damage (stomach, *P* < 0.001; thyroid, *P* < 0.001) (Figure 6A,B), led to significantly milder body weight loss (PBS/^211^At-anti-HER2 mAb (ATE) vs SP/^211^At-anti-HER2 mAb (ATE), *P* < 0.001) (Figure 4B), accompanied by a more rapid recovery from the side effects (*P* = 0.002) (Figure 4C). Relieving gastric damage and/or thyroid dysfunction via NIS inhibition may result in mild toxicity after ^211^At-RIT. Oh et al. demonstrated an optimized protocol for establishing an athyroid mouse model using iodide-131 (^131^I) ablation.^47^ Although whether ^131^I ablation causes body weight loss depends on the administered radioactivity,^48^ athyroid mice established using the optimized protocol showed thyroid dysfunction, such as low serum T4 hormone levels and attenuated iodine-125 (^125^I) uptake in the thyroid without a loss of body weight. Histological analysis showed that follicles were not observed in the thyroid tissues collected from the athyroid mouse model,^47^ indicating more severe damage than that in the thyroid after ^211^At-RIT (Figure 7). That is, thyroid dysfunction in the mouse model with more severe thyroid damage than that induced by ^211^At-RIT did not cause body weight loss. Thus, milder alterations in the thyroid after ^211^At-RIT do not seem to cause weight loss. Although toxicity after ^211^At-RIT must occur complexly, considering these findings, relief of gastric damage, but not thyroid dysfunction, might contribute to milder body weight loss following ^211^At-RIT with NIS inhibition.

In the phase 1 study for [^211^At]­NaAt, dose-limiting toxicities consisted of hematologic toxicities—specifically grade 3 lymphopenia and leukopenia—observed in patients who received 3.5 MBq/kg.^49^ In addition to these dose-limiting toxicities, adverse events related to the physiological accumulation of ^211^At in the salivary glands and gastrointestinal tract, such as xerostomia, nausea, vomiting, and decreased appetite, were reported.^49^ Gastrointestinal symptoms were generally grades 1 or 2 and typically resolved within 2–3 days, although in some cases they persisted for more than 1 week.^49^ In ^211^At-targeted alpha therapy, symptoms from the physiological accumulation of free ^211^At in the thyroid, salivary glands, and gastrointestinal tract should be carefully prevented to ensure treatment with minimal toxicity to patients with cancer. Competitive inhibition of NIS may have clinical potential to reduce these adverse events in ^211^At-RIT.

## 5 Conclusions

Combining SA protection to avoid ^211^At-induced antibody denaturation and competitive inhibition of NIS with SP to reduce ^211^At uptake in the stomach and thyroid glands is feasible and broadens the therapeutic window. This combined strategy facilitates the clinical application of ^211^At-RIT in cancer treatment.

## Supplementary Material



## Notes

Materials and methods for toxicity test and histological examination; RBC count and Hb levels after ^211^At-RIT under competitive inhibition of NIS by SP (Figure S1); and histological findings in the large intestine and spleen 35 days after ^211^At-RIT (Figures S2 and S3) (PDF)

## Abbreviations

ALT: alanine aminotransferase
ANOVA: a repeated-measures analysis of variance
AST: aspartate aminotransferase
^211^At: astatine-211
ATE: *N*-succinimidyl-3-(trimethylstannyl)­benzoate
BUN: blood urea nitrogen
CPM: counts per minute
Cre: creatinine
DSB: double-strand break
EPR effect: enhanced permeability and retention effect
Fab: fragment antigen-binding
FT: flow-through
Hb: hemoglobin
HER2: human epidermal growth factor receptor 2
HSA: human serum albumin
LDH: lactate dehydrogenase
mAb: monoclonal antibody
MSB: *N*-[2-(maleimido)­ethyl]-3-(trimethylstannyl)­benzamide
NIS: sodium iodide symporter
PBS: phosphate-buffered saline
PLT: platelet
PODS: phenyloxadiazolyl methylsulfone
RBC: red blood cell
RI: radioisotope
RIT: radioimmunotherapy
ROS: reactive oxygen species
RT: room temperature
SA: sodium ascorbate
sdAb: single-domain antibody
SP: sodium perchlorate
SPECT: single-photon emission computed tomography
WBC: white blood cell.
