# Supplementary material for: Attenuated Toxicity and Antitoxic Mechanism via Sodium Iodide Symporter Inhibition-Based Tumor-Selective Delivery in Astatine-211 Radioimmunotherapy
Source: Mol Pharm. 2026 Mar 1;23(4):2422–35. doi: 10.1021/acs.molpharmaceut.5c01438 (PMC13058875; doi:10.1021/acs.molpharmaceut.5c01438)
Supplement: Supplementary file 1 [file mp5c01438_si_001.pdf]

Supplementary information for:

Attenuated Toxicity and Antitoxic Mechanism via Sodium Iodide Symporter Inhibition-Based Tumor-Selective Delivery in Astatine-211 Radioimmunotherapy

Hiroki Takashima[a], Ryo Tsumura[a], Yoshikatsu Koga[a], Takahiro Anzai[a], Xiaojie Yin[b], Nozomi Sato[b], Yudai Shigekawa[b], Yousuke Kanayama[b], Akihiro Nambu[b], Sachiko Usuda[b], Hiromitsu Haba[b], Shingo Sakashita[c], Anri Inaki[d], Shino Manabe[e,f,g], and Masahiro Yasunaga[a,*]

[a]Division of Developmental Therapeutics, Exploratory Oncology Research & Clinical Trial Center, National Cancer Center, 6-5-1 Kashiwanoha, Kashiwa, Chiba 277-8577, Japan.

[b]Nishina Center for Accelerator-Based Science, RIKEN, 2-1 Hirosawa, Wako, Saitama 351-0198, Japan.

[c]Division of Pathology, Exploratory Oncology Research & Clinical Trial Center, National Cancer Center, 6-5-1 Kashiwanoha, Kashiwa, Chiba 277-8577, Japan.

[d]Division of Functional Imaging, Exploratory Oncology Research & Clinical Trial Center, National Cancer Center, 6-5-1 Kashiwanoha, Kashiwa, Chiba 277-8577, Japan.

[e]Laboratory of Synthetic Biomolecular Chemistry, School of Pharmacy and Pharmaceutical Sciences and Institute of Medical Chemistry, Hoshi University, 2-4-41 Ebara, Shinagawa-ku, Tokyo 142-8501, Japan.

[f]Research Center for Pharmaceutical Development, Graduate School of Pharmaceutical Sciences & Faculty of Pharmaceutical Sciences, Tohoku University, 6-3 Aoba, Aramaki, Aoba-ku, Sendai 980-8578, Japan.

[g]Glycometabolic Biochemistry Laboratory, RIKEN, 2-1 Hirosawa, Wako, Saitama 351-0198, Japan.

*Email: mayasuna@east.ncc.go.jp; Tel: +81-4-7134-6857; Fax: +81-4-7134-6866

1. MATERIALS AND METHODS

1.1 Toxicity Test. Mice bearing OE19 subcutaneous tumors were intraperitoneally administered 1.2 μmol/g sodium perchlorate (SP) (Sigma-Aldrich, St. Louis, MO, USA) dissolved in phosphate-buffered saline (PBS) at 1 and 24 h before radioimmunotherapy (RIT) with 1 MBq of astatine-211-labeled trastuzumab conjugated with *N*-succinimidyl-3-(trimethylstannyl)benzoate (^{211}At-anti-HER2 mAb (ATE)). Blood was collected via cardiocentesis from the mice under isoflurane anesthesia before treatment (non-treatment group) and at 1, 3, 7, and 10 days after RIT. A Celltac α (NIHON KOHDEN, Tokyo, Japan) was used for complete blood count analyses.

1.2 Histological Examination. Mice bearing OE19 subcutaneous tumors were administered 1 MBq ^{211}At-anti-HER2 mAb (ATE) after pretreatment with either PBS or SP. Before the treatment (non-treatment group), as well as at 35 days after ^{211}At-RIT, the mice were euthanized. The normal organs were excised and fixed with 10% formalin neutral buffer solution (Muto Pure Chemicals, Tokyo, Japan) overnight at 4 °C and embedded in paraffin (Sakura Finetek, Tokyo, Japan). Tissue sections (3 μm thick) were visualized using hematoxylin and eosin staining. To evaluate cellular infiltration into the red pulp in the spleen tissues, we extracted eosin-positive and hematoxylin-negative areas, which were defined as red pulp without cellular infiltration, using the Hybrid Cell Count in the BZ-X800 Analyzer software (Keyence, Osaka, Japan). The percentage of red pulp without cellular infiltration was calculated by dividing the extracted eosin-positive and hematoxylin-negative areas by the total area of the tissue. Two fields of view per tissue section were analyzed.

1.3 Statistical Analysis. One-way analysis of variance (ANOVA) was used to analyze the red blood cell (RBC) count and hemoglobin (Hb) levels. Welch's one-way ANOVA, followed by the Games-Howell post-hoc test, was used to analyze eosin-positive and hematoxylin-negative areas in the spleen tissues. Statistical analyses were performed using SPSS statistical software version 29 (IBM, Armonk, NY, USA), and $P < 0.05$ was considered statistically significant.

2. RESULTS

2.1 RBC Count and Hb Value after ^{211}At-RIT under Competitive Inhibition of Sodium Iodide Symporter with SP. Under competitive inhibition of sodium iodide

symporter (NIS) by SP, ^{211}At-RIT did not cause a significant decrease in RBC count or Hb levels (RBC, $P = 0.257$; Hb, $P = 0.142$) (Figure S1).

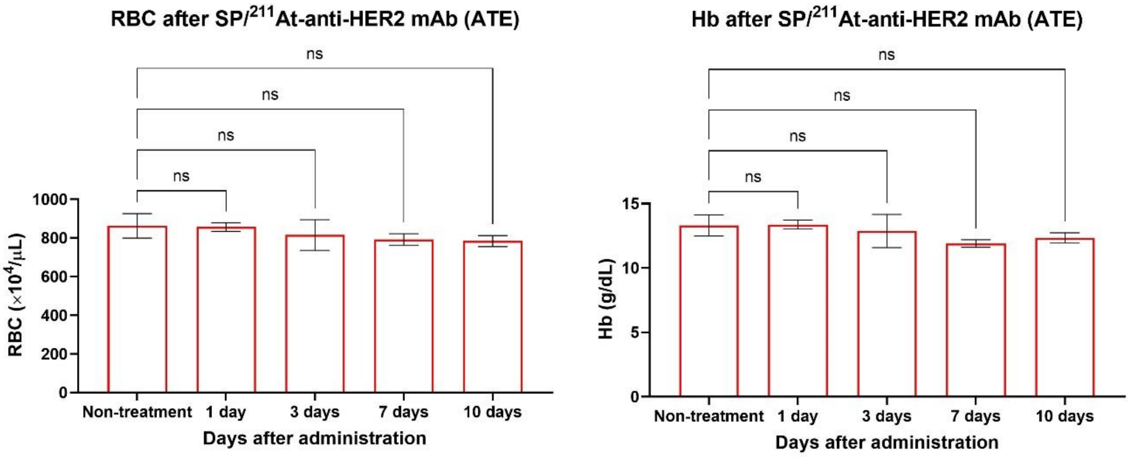

Figure S1. RBC count and Hb levels after ^{211}At-RIT under competitive inhibition of NIS by SP. Mice bearing OE19 tumors were administered SP plus 1 MBq ^{211}At-anti-HER2 mAb (ATE). Blood was collected before treatment and at 1, 3, 7, and 10 days after ^{211}At-RIT, and the RBC count and Hb levels were evaluated ($n = 3$). Data are shown as mean ± standard deviation.

2.2 Histological Findings in Large Intestine. No differences were observed in the large intestine findings 35 days after ^{211}At-RIT between the non-treated, PBS plus ^{211}At-anti-HER2 mAb (ATE), and SP plus ^{211}At-anti-HER2 mAb (ATE) groups (Figure S2).

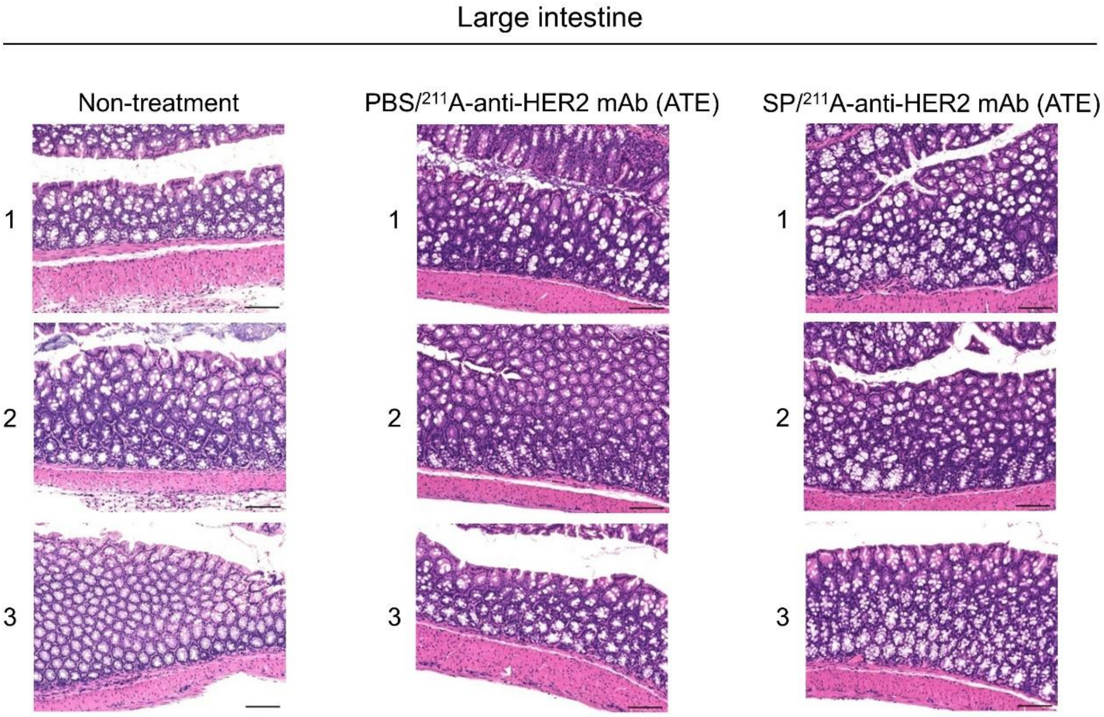

Figure S2. Histological findings in the large intestine 35 days after ^{211}At-RIT. The large intestine was visualized using hematoxylin and eosin staining. No differences were observed in the findings of the large intestine between the nontreated, PBS/^{211}At-anti-HER2 mAb (ATE), and SP/^{211}At-anti-HER2 mAb (ATE) groups. Three mice from each group were examined for each parameter. Scale bar, 100 µm.

2.3 Histological Findings in Spleen. The spleen was histologically examined 35 days after 1 MBq ^{211}At-anti-HER2 mAb (ATE) administration. Compared with the tissues collected from the non-treatment group, the percentage of red pulp without cellular infiltration was significantly lower in the groups treated with PBS plus ^{211}At-anti-HER2 mAb (ATE) or SP plus ^{211}At-anti-HER2 mAb (ATE) (non-treatment vs PBS/^{211}At-anti-HER2 mAb (ATE), P = 0.020; non-treatment vs SP/^{211}At-anti-HER2 mAb (ATE), P = 0.021) (Figure S3A–C). Conversely, this finding suggests increased cellular infiltration into the red pulp after ^{211}At-RIT, implying an inflammatory response in the hematopoietic system after radiation exposure.[1] In contrast, there was no difference in histological findings between the groups treated with PBS plus ^{211}At-anti-HER2 mAb (ATE) and SP plus ^{211}At-anti-HER2 mAb (ATE) (Figure S3A–C).

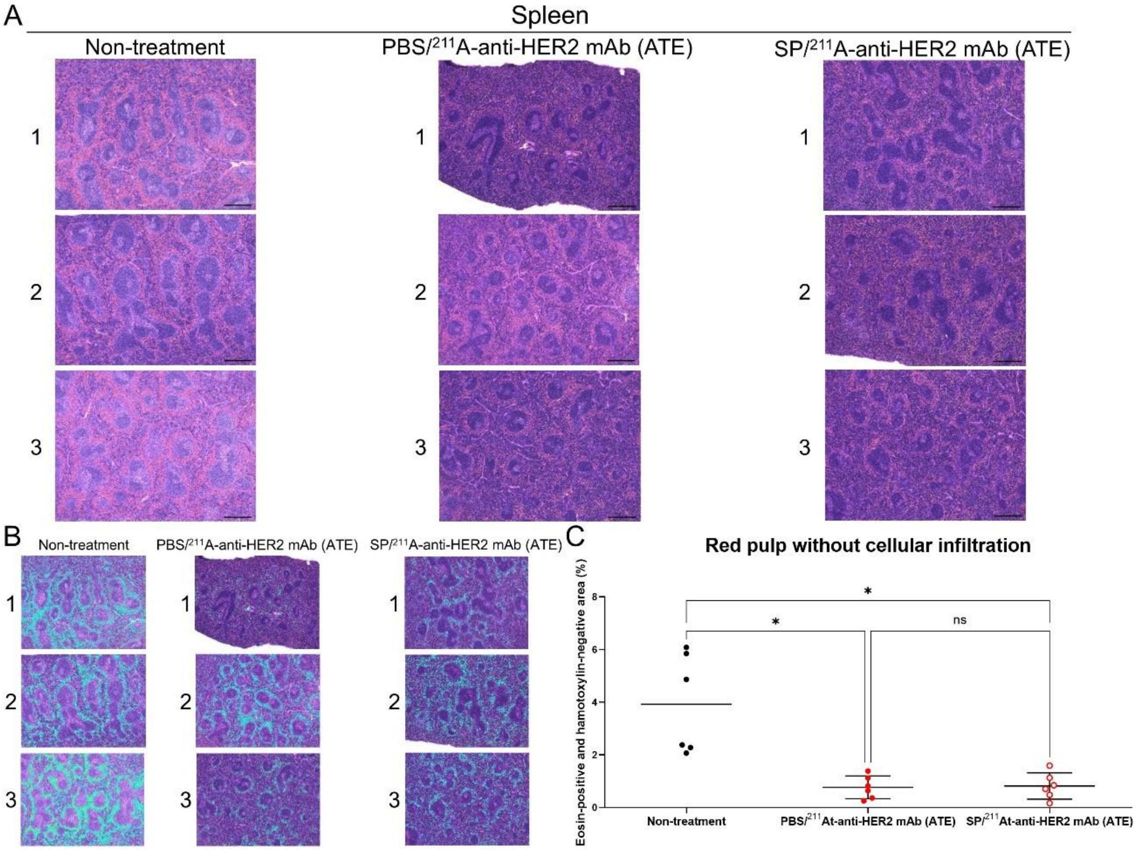

Figure S3. Histological findings in the spleen 35 days after ^{211}At-RIT. (A) Hematoxylin and eosin staining. Three mice from each group were examined. Scale bar, 500 µm. (B) Extraction of eosin-positive and hematoxylin-negative areas, defined as red pulp areas without cellular infiltration. Three mice from each group were examined. (C) The percentage of red pulp without cellular infiltration in the groups treated with PBS plus ^{211}At-anti-HER2 mAb (ATE) or SP plus ^{211}At-anti-HER2 mAb (ATE) was significantly lower than that in the non-treatment group (non-treatment vs PBS/^{211}At-anti-HER2 mAb (ATE), $P = 0.020$; non-treatment vs SP/^{211}At-anti-HER2 mAb (ATE), $P = 0.021$). No significant differences were observed between the groups treated with PBS plus ^{211}At-anti-HER2 mAb (ATE) and SP plus ^{211}At-anti-HER2 mAb (ATE) ($P = 0.977$). Three mice from each group were examined. Two fields of view per tissue section were analyzed. *, $P < 0.05$.

References

(1) Lorimore, S. A.; Coates, P. J.; Scobie, G. E.; Milne, G.; Wright, E. G. Inflammatory-type responses after exposure to ionizing radiation *in vivo*: a mechanism for radiation-induced bystander effects? *Oncogene* **2001**, *20*, 7085-7095.
